# Bio-nanopore technology for biomolecules detection

**DOI:** 10.1007/s44307-024-00051-7

**Published:** 2024-12-06

**Authors:** Peizhi Li, Dan Liang, En Yang, Mustafa Zeb, Huiqi Huang, Haihui Sun, Wenhan Zhang, Chifang Peng, Yuan Zhao, Wei Ma

**Affiliations:** 1 School of Food Science and Technology, State Key Laboratory of Food Science and Resources, Jiangnan University, Wuxi, 214122 Jiangsu China; 2 Key Laboratory of Synthetic and Biological Colloids, Ministry of Education, School of Chemical and Material Engineering, Jiangnan University, Wuxi, 214122 Jiangsu China; 3 Yichun Dahaigui Life Science Co., Ltd, Yichun, Jiangxi 336000 China; 4 Wuxi Gujing Biotechnology Co., Ltd, Wuxi, Jiangsu China; 5 Gao’an Qinghe Oil and Fat Co., Ltd, Gao’an, Jiangxi 330800 China

**Keywords:** Bio-nanopore, DNA sequencing, RNA sequencing, Protein detection, Small molecule detection

## Abstract

Bio-nanopore technology holds great promise in biomacromolecule detection, with its high throughput and low cost positioning it as an ideal detection tool. This technology employs a unique detection mechanism that utilizes nanoscale pores to rapidly and sensitively convert biological molecules interactions into electrical signals, enabling real-time, single-molecule detection with exceptional sensitivity. This review focuses on the latest advancements in this technology across various domains, including DNA and RNA sequencing, protein detection, and small molecule identification. Additionally, future trends are explored, providing a comprehensive and in-depth perspective on the role of bio-nanopore technology in biomolecule detection.

## Introduction

As biotechnology continues to evolve, bio-nanopore technology is gaining significant attention as an emerging detection method. This innovative approach generates characteristic current pulse signals by applying a voltage to facilitate the passage of analytes through nanopores. By analyzing the variations in current blockage and the duration of these blockages as substances traverse the nanopore, researchers can infer the physicochemical properties and structural characteristics of the analytes. Bio-nanopore technology is particularly well-suited for applications in genomics, transcriptomics, and clinical diagnostics, owing to its notable advantages, including low cost, high throughput, and label-free detection (Dorey, A. & Howorka, S., 2024).

In recent years, the application of bio-nanopore technology has achieved remarkable advancements. This technology is now widely employed in DNA and RNA sequencing, peptide analysis, protein detection, and small molecule identification. For instance, bio-nanopore sequencing technology enables the accurate determination of genomic sequences and facilitates the identification of epigenetic modifications, thereby enhancing our understanding of the complexity and diversity of biological samples. However, despite the distinct advantages of bio-nanopore technology in terms of detection accuracy and application range, it continues to face several challenges, including low detection capability and stability, inadequate detection efficiency and throughput, as well as interference from signal noise. Therefore, conducting in-depth research and optimizing the detection performance of bio-nanopore technology will unlock broader prospects for future bioscience research and clinical applications.

**Fig. 1:**
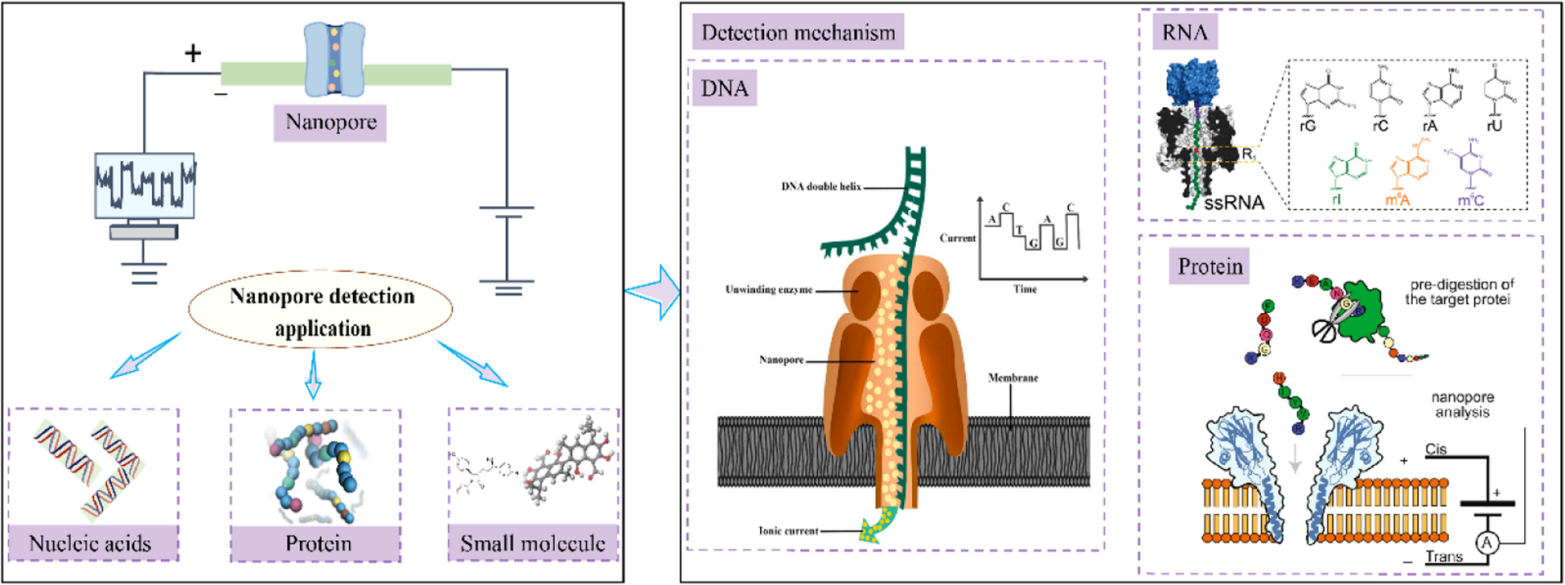
Application of bio-nanopore detection technology in biomolecular detection. DNA: The principle of the MinION Oxford nanopore sequencer involves the unwinding of DNA by an enzyme at the sequencer's center, allowing single-stranded DNA to be fed into a solid-state nanopore (Shivashakarappa, K. et al., 2022). RNA: Individual RNA base recognition in immobilized oligonucleotides using a protein nanopore (Ayub, M. & Bayley, H., 2012). Protein: Peptides were pre-hydrolyzed by specific proteases (e.g. trypsin) and the resulting peptides were measured upon peptide translocation to the nanopore (Lucas, F. L. R. et al., 2021)

This review systematically analyzes the recent advancements in bio-nanopore technology within the realm of biomolecule detection (Fig. 1). By focusing on research progress associated with novel and emerging technologies across various assay applications, we aim to provide new insights and comprehensive analyses to foster the continued development and innovation of nanopore technology.

## Nucleic acids

Bio-nanopore technology has found extensive applications in nucleic acid detection and sequencing, enabling the effective detection of trace amounts of nucleic acid molecules in various samples. This includes identifying disease-related markers such as miR-21, miR-122, miR-155, and miR-182. In recent years, the application of bio-nanopore technology in nucleic acid sequencing has remained a prominent research topic, with researchers continually pursuing technological innovations to broaden its scope of applications. Consequently, this paper will focus on the technological advancements of bio-nanopores in nucleic acid sequencing.

**Fig. 2:**
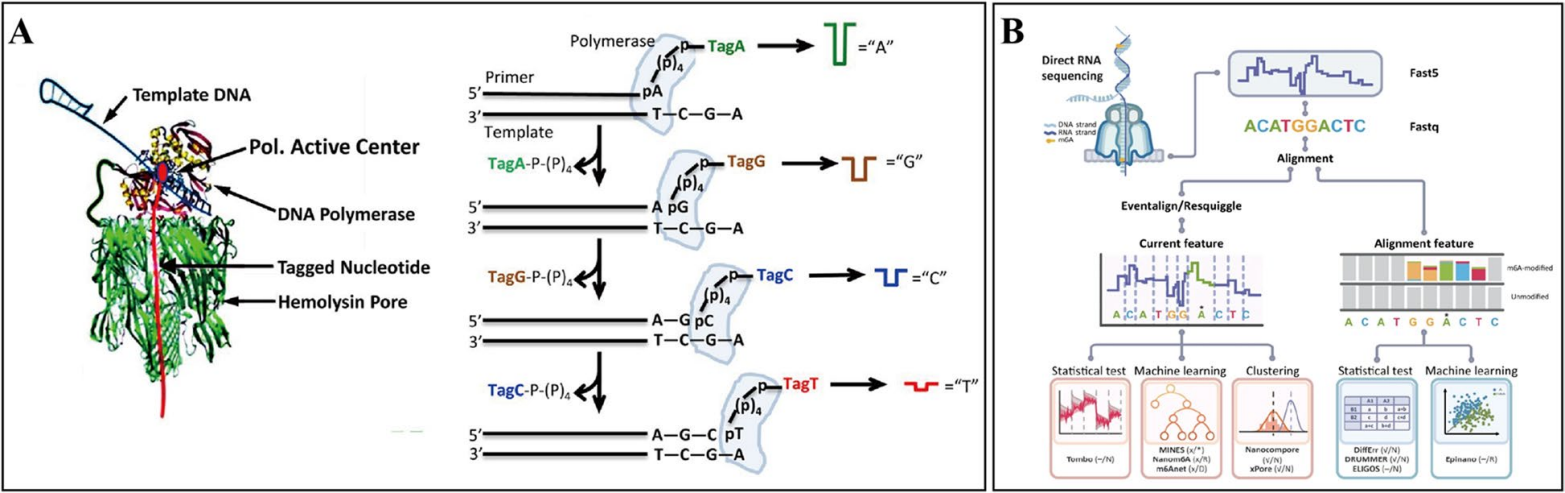
Application of bio-nanopore detection technology in nucleic acids. **A** The synthetic principles of nanopore DNA sequencing (Fuller, C. W. et al., 2016). **B** Strategies and tools used for m6A mapping on the direct RNA sequencing platform based on Oxford Nanopore Technologies (Zhong, Z.-D. et al., 2023)

In the context of nucleic acid sequencing, nanopore proteins and their associated motor proteins are pivotal. Bio-nanopore nucleic acid sequencing utilizes engineered protein pores that precisely regulate critical properties such as size, charge, hydrophobicity, and polarity. These attributes are essential for maintaining pore stability and optimizing the signal-to-noise ratio during sequencing. A significant advancement in enhancing signal-to-noise ratios involves the development of synthetic motor enzymes that slow the movement of single-stranded DNA (ssDNA) through the nanopore (Fig. 2A) (Fuller, C. W. et al., 2016), allowing for nucleotide-by-nucleotide processing. In recent years, significant advancements in DNA sequencing technology have been made, exemplified by Oxford Nanopore Technologies' (ONT) MinION device, with the R9.4 version achieving an accuracy of 94% and the R10 version further improving it to 99.994% (Dorey, A. & Howorka, S., 2024). The R9 version utilized the E. coli CsgG pore, while the R10 version introduced a dual-reader design, though specific information about R10 remains scarce. The Oxford Nanopore R10.4.1 microarray produces high-quality 16S rRNA data, enabling species-level analyses of microbial communities that were challenging to achieve with previous technologies. This chip significantly enhances the accuracy of microbial community analysis and serves as a powerful tool for research in environmental microbiology. Additionally, the R10.4.1 chip offers the advantage of generating more reads while being more cost-effective compared to other platforms (Zhang, T. et al., 2023). The R10.4 flow cell demonstrates a modal read accuracy of 99.1% or higher in single-cell whole-genome amplification (scWGA) and whole-genome sequencing, marking a substantial improvement over the R9.4.1 version (Ni, Y. et al., 2023). This high accuracy is crucial for the precise analysis of cancer cell genomes, particularly in cancer cytogenetics. The technological upgrade from R9.4 to R10 has greatly improved both the precision of genome assembly and the sensitivity of variant detection. By enhancing sequencing accuracy, this upgrade has reduced errors and uncertainties, resulting in a more efficient research process. Scientists can now analyze complex genome structures more effectively, discover new genetic variants, and gain deeper insights into how these variants are related to diseases. Furthermore, these technological innovations have led to the emergence of new research paradigms, such as employing long-read data for genetic disease screening and drug-target identification, which opens up exciting opportunities for the advancement of precision medicine and individualized therapies. ONT has fostered innovation in nanopore sequencing technology by investigating various enzymes, including mutated ATP-driven helicases. The adoption of nanopore DNA sequencing is increasing across various applications, making it even more prevalent in genomic testing. A programmable nanopore sequencing technology allows for the simultaneous detection of multiple pathogenic short tandem repeat sequences associated with tandem repeat amplification, enhancing the precision and personalization of the assay (Stevanovski, I. et al., 2022). The compact and portable MinION device has been successfully deployed during outbreaks of Ebola, Zika, and SARS-CoV-2. Recent research has also led to the creation of open-source software designed for flexible multiplex PCR targeting user-defined genes or regions within the *P. falciparum* genome (de Cesare, M. et al., 2024), significantly broadening the scope for genomic data collection using nanopore sequencing.

Bio-nanopore technology is also applicable to RNA sequencing. Traditional methods necessitate the reverse transcription of RNA to synthesize complementary DNA (cDNA) strands, creating RNA-cDNA hybrid double-stranded structures before sequencing. However, in recent years, significant advancements have been made in the technology for directed RNA sequencing (DRS). This innovative approach eliminates the traditional reverse transcription step and employs the unique current signals generated as the RNA strand passes through a nanopore to directly determine its sequence. This method reduces time and operational complexity compared to reverse transcription-dependent cDNA sequencing techniques and preserves the original information of the RNA, including its critical modification states and complex structural features. Recently, Oxford Nanopore introduced the new RNA004 kit, which achieves a 3- to fourfold increase in sequencing output compared to its predecessor, RNA002. It maintains an overall read accuracy of 93.5%, an improvement over RNA002’s 92.1%. Furthermore, the RNA004 kit shows enhanced sensitivity in detecting a wider variety of RNA modifications (Liu-Wei, W. et al., 2024). Current research is increasingly focused on identifying and studying the functional roles of RNA modifications. Oxford Nanopore Technologies-based DRS can be used to detect N6-methyladenine (m6A) modifications (Fig. 2B) (Zhong, Z.-D. et al., 2023). By exploring novel nanopore proteins, significant strides have been made in detecting both known and unknown modifications in natural RNA. For instance, by constructing high-resolution Mycobacterium pubescens membrane protein A (MspA) nanopores, multiple nucleotide modifications have been successfully identified and differentiated (Wang, Y. et al., 2022), detecting up to seven common modifications simultaneously. These include 5-methylcytidine (m5C), m6A, N7-methylguanosine (m7G), N1-methyladenosine (m1A), inosine (I), pseudouridine (Ψ), and dihydrouracil (D). Nano-tRNAseq is a specialized RNA sequencing technique built on Oxford nanopore technology that utilizes fluctuations in current signals to identify nucleotide sequences in RNA molecules as they pass through the nanopore. Researchers have achieved accurate tRNA sequencing by reprocessing the raw nanopore current intensity signals and incorporating RNA junctions to fill the 5' and 3' ends of tRNAs (Lucas, M. C. et al., 2024), thereby enhancing base calling and mappability for these molecules. The innovative deep learning framework TandemMod combines nanopore sequencing technology with advanced deep learning algorithms, enabling the efficient and accurate identification of multiple RNA modifications within a single sample. A significant advantage of this technology is its capacity to resolve various modification types simultaneously (Wu, Y. et al., 2024) providing unprecedented depth and breadth for epitranscriptome research. DRS library preparation is a crucial step in the identification of RNA modifications. High-quality library preparation maximizes the retention of the modification states of RNA molecules and minimizes the loss or alteration of modification information during the library construction process, thereby enhancing the accuracy and reliability of modification identification (Jain, M. et al., 2022). Different DRS library preparation kits exhibit significant variations in their ability to protect RNA modifications, improve sequencing efficiency, and ensure compatibility. Consequently, selecting the appropriate kit is essential for obtaining high-quality sequencing data and accurate modification identification results. While some kits, such as RNA002, may produce higher-depth sequencing data, they also come with a correspondingly higher cost. When RNA kits are employed for RNA modification detection, the TandemMod model can serve as a powerful analytical tool to help interpret the sequencing data generated by these kits and identify various modification types on the RNA molecule. Although there is currently no direct evidence that the TandemMod model reduces the cost of generating high-depth data, utilizing this model can lead to faster and more accurate analysis results for the same volume of data. This efficiency can reduce the time and computational resources required for data processing and ultimately lower the overall costs of subsequent data analysis and interpretation. In addition, single-cell RNA sequencing technology has shown remarkable advantages used in the tumor immune microenvironment (Li, P.-H. et al., 2022). It enables high-resolution analysis of cellular heterogeneity, reveals the functional status and molecular characteristics of various immune cell subpopulations, and facilitates the discovery of new immune cell types and tumor-associated gene expression patterns. These insights can lead to the identification of potential targets and therapeutic strategies for tumor immunotherapy.

While bio-nanopore sequencing technology holds significant promise in the field of nucleic acid sequencing, it continues to encounter several challenges. In the field of DNA and RNA sequencing, in response to the challenges of enzyme dependency, limited throughput, and high background noise, future research will innovate in the following three areas: 1) Achieving enzyme-free sequencing: cutting-edge gene editing tools such as CRISPR-Cas9 will be employed to precisely mutate nanopore proteins, such as MspA, to eliminate enzyme binding sites. This strategy will be complemented by specific chemical modifications using metal ions (e.g., Ni^2^⁺, Hg^2^⁺) or organic small molecules to enhance the stability of interactions between the nanopore and nucleic acid molecules, thus fully realizing enzyme-free sequencing. Additionally, optimizing the electric field driving system will enable precise regulation of electric field parameters, ensuring the efficient and directional traversal of negatively charged nucleic acid molecules, thereby facilitating significant advancements in enzyme-free sequencing technology. 2) Enhancing sequencing throughput: the refinement of micro-nanofabrication technology will allow for the construction of high-density, uniformly distributed nanopore arrays on chip surfaces, significantly increasing sequencing throughput. Furthermore, combining computational simulations with experimental validations will enable the optimal design of nanopore geometries (such as conical or funnel-shaped) to accelerate the passage of nucleic acids while maintaining high signal resolution. This dual optimization strategy will enhance both sequencing speed and accuracy. 3) Reducing background noise: to minimize background noise, it is essential to select and purify biological nanopore materials—such as MspA protein—stringently, ensuring high purity and low defect levels to reduce non-specific binding. Additionally, fine-tuning environmental parameters during sequencing, including temperature, pH, and ion concentration, will help mitigate environmental interference, ensuring clarity and reliability in sequencing results.

## Protein

**Fig. 3:**
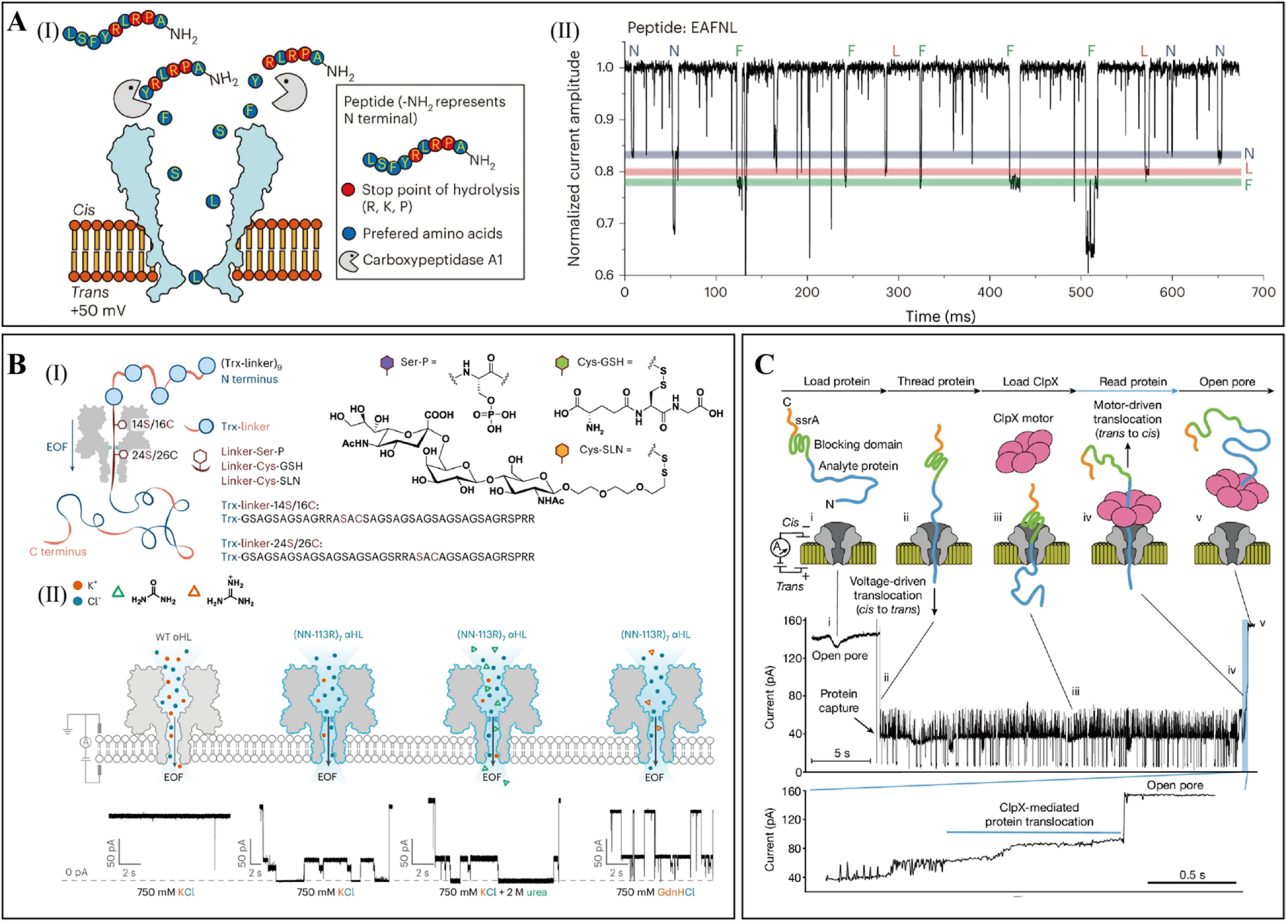
Application of bio-nanopore detection technology in protein. **A** Real-time identification of amino acids during peptide hydrolysis. **a** Schematic representation of the assay. Peptides and Carboxypeptidase A1 are introduced directly into the nanopore. Individual amino acids (excluding arginine (Arg), lysine (Lys), and proline (Pro)) can be cleaved from the peptide chain and detected. **b** Representative current traces of amino acid signals during peptide hydrolysis. Target amino acids can be accurately identified based on normalized current amplitudes (Zhang, M. et al., 2024). **B** Enzyme-free nanopore detection of post-translational modifications within long peptides. **a** Detection of post-translational modifications in protein linkers driven through nanopores using electroosmotic flow technology. **b** Electroosmotic transport of thioredoxin junction octamers through the nanopore, facilitated by the dissociation solution (Martin-Baniandres, P. et al., 2023). **C** Reading nanopore proteins using defolding enzymes (Motone, K. et al., 2024)

In recent years, with the continuous optimization and innovation of bio-nanopore technology, the research hot topic is gradually focusing on single-molecule protein sequencing and peptide detection. For instance, engineered hetero-octameric mycobacterium sporophorum protein A (MspA) nanopores, modified with a single Ni^2^⁺ ion, can recognize 20 protein amino acids along with four modified amino acids: Nω, N'ω-dimethylarginine (Me-R), O-acetylthreonine (Ac-T), N4-(β-N-acetyl-D-glucosamine) asparagine (GlcNAc-N), and O-phosphoserine (PS) (Wang, K. et al., 2024). With the support of machine learning, this nanopore sensor achieved an impressive accuracy of 98.6% in distinguishing these amino acids and their modifications, marking a significant milestone in the use of nanopore technology for protein sequencing and proteomics research. Recent studies have further showcased the capability to detect cleaved amino acids in real-time during peptide hydrolysis, opening new avenues for inferring peptide sequences. A copper(II)-functionalized MspA nanopore with an N91H substitution was able to identify all 20 protein amino acids, two amino acids with post-translational modifications (PTMs) (phosphoserine and acetyl-lysine), and one unnatural amino acid (CMC) (Fig. 3A) (Zhang, M. et al., 2024), achieving a validation accuracy of 99.1%. Moreover, nanopore technology has enabled high-precision detection of phosphorylated post-translational modifications in peptide chains at the single-molecule level, eliminating the need for complex labeling and overcoming the limitations of traditional methods. By linking peptides to DNA oligonucleotides to create peptide-oligonucleotide conjugates (POCs), peptide chains can pass through the nanopore with the assistance of DNA kinase, facilitating the successful detection of phosphorylation modifications in individual peptide chains (Nova, I. C. et al., 2024). The integration of bio-nanopore technology with host–guest chemistry enables the identification and sequence determination of peptide chains on an amino acid-by-amino acid basis. This is achieved by designing specific peptide probes that interact with the host–guest complexes within the nanopore (Zhang, Y. et al., 2024). Enzymatic reaction-free nanopore detection allows for the precise identification of post-translational modifications in long polypeptide chains (Fig. 3B) (Martin-Baniandres, P. et al., 2023). This technology employs nanopore sensors to directly target specific modification sites within the peptide chain, eliminating the need for complex pre-treatment steps or enzymatic reactions. By monitoring the current changes that occur as the peptide chain passes through the nanopore, researchers can efficiently and rapidly determine both the type and location of modifications. This method enhances detection sensitivity and provides a valuable new tool for investigating the functions and regulatory mechanisms of protein post-translational modifications. The integration of nanopore technology with the unfolding enzyme ClpX allows for high-precision sequencing of long protein chains at the single-molecule level with the capability of multiple re-reads. This innovative approach is adept at identifying mutations in individual amino acids, as well as detecting post-translational modifications (PTMs) such as phosphorylation and glycosylation on protein chains, showcasing exceptional sensitivity and accuracy. This advancement establishes a solid foundation for full-length protein identification, achieving the highest level of resolution in protein forms (Fig. 3C) (Motone, K. et al., 2024).

Bio-nanopore technology presents significant potential in the realm of protein analysis; however, it continues to face numerous challenges. To address these obstacles and facilitate further advancements in the technology, future research should prioritize the following areas: 1) Enhancement of sequencing accuracy and resolution: the performance of sequencing can be improved by integrating molecular motors with electroosmotic technology. The incorporation of molecular motors allows for the active regulation of protein molecule transit speed within nanopores, effectively slowing their movement and thereby extending the signal acquisition time, which ultimately enhances sequencing accuracy. Additionally, by employing electroosmotic technology, electric field forces can be utilized to guide protein molecules directionally through the nanopores, minimizing randomness and significantly improving sequencing resolution. 2) Coping with protein diversity and complexity: by employing molecular dynamics simulations alongside single-molecule stretching experiments, researchers can systematically analyze the interactions between proteins and nanopores. The integration of simulation predictions with experimental validation will facilitate the continuous optimization of nanopore designs, thereby improving the capability to resolve complex protein samples. 3) Enhancement of simultaneous detection of multiple proteins: the development of a high-throughput parallel detection platform that integrates multiple nanopore arrays is a crucial step towards achieving this goal. Advanced micro-nanofabrication techniques can be employed to precisely assemble multiple nanopore arrays on a chip, using materials such as graphene or silicon nitride with excellent conductivity as the nanopore substrate to enhance detection efficiency and stability. This platform will enable the parallel processing of multiple samples or the simultaneous detection of various proteins, significantly boosting detection efficiency. Additionally, incorporating multiple detection modalities—such as combinations of electrochemical, optical, and mass spectrometry techniques—will help validate and complement the results obtained from nanopore assays (Yu, Y. et al., 2024). Techniques such as cyclic voltammetry, differential pulse voltammetry, and electrochemical impedance spectroscopy can be effectively integrated with optical and mass spectrometry methods to facilitate comprehensive analysis of protein molecules traversing the nanopores, providing valuable insights into their delivery properties, binding kinetics, and structural changes. Incorporating these multiple detection modalities will help to improve the understanding of protein behavior, thereby advancing the sensitivity and specificity of biomolecular detection.

## Small molecule

**Fig. 4:**
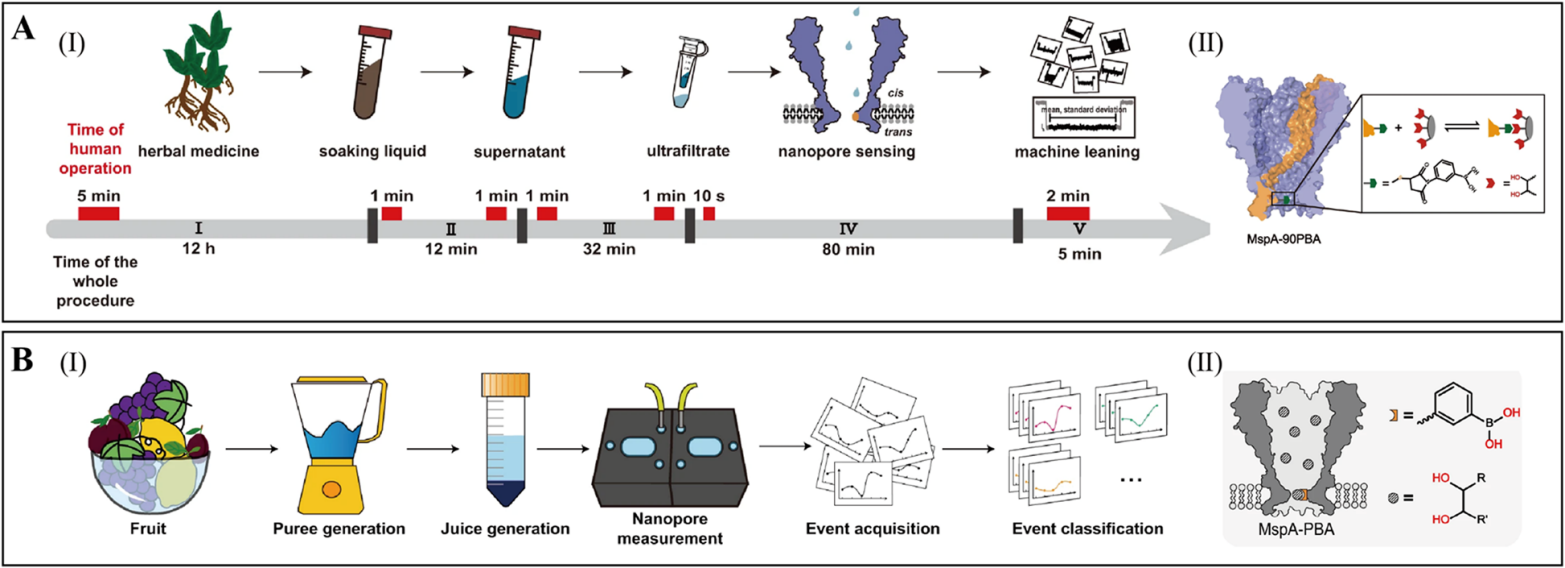
Application of bio-nanopore detection technology in small molecule. **A** Nanopore analysis of salvianolic acid in herbal medicine. **a** Nanopore workflow for the identification of salvianolic acid directly from natural herbs. **b** Mechanism of MspA-90PBA nanopore sensing of salvianolic acid (Fan, P., Zhang, S., et al., 2024). **B** Nanopore analysis of cis-diols in fruits. **a** Schematic of rapid analysis of natural fruit juices. **b** Phenylboronic acid-modified nanopore sensor (Fan, P., Cao, Z., et al., 2024)

Bio-nanopore perforation assay research also holds significant potential for small molecule analysis. For instance, a single phenylboronic acid-modified MspA nanopore (MspA-90PBA) has successfully identified and quantitatively analyzed various salvianolic acid compounds found in traditional Chinese herbal medicine (Fig. 4A) (Fan, P., Zhang, S., et al., 2024). This innovation not only streamlines the sample pretreatment process but also significantly enhances the accuracy and efficiency of assays, providing a valuable technological tool for quality control in herbal medicine and research on active ingredients. Furthermore, MspA-PBA has demonstrated the capability to accurately identify a diverse range of cis-diols in fruits, including 1,2-diphenols, alditols, α-hydroxy acids, and sugars (Fig. 4B) (Fan, P., Cao, Z., et al., 2024). With the aid of machine learning algorithms, this sensor achieves highly accurate detection of cis-diols in complex fruit samples while significantly simplifying the sample pretreatment process. These advancements underscore the technological innovation and broad application potential of nanopore analysis in identifying components within complex biological samples.

Compared to mass spectrometry, liquid chromatography, and gas chromatography, nanopore technologies typically offer greater portability, lower analytical costs, simplified sample preparation processes, and the capability to simultaneously detect multiple analytes, making them particularly well-suited for portable food analysis. However, future research should focus on the following areas: 1) Development of efficient sample pre-treatment techniques: emphasis should be placed on creating sample pre-treatment methods, such as solid phase extraction, membrane filtration, or selective precipitation, to eliminate complex interfering substances—like fats, sugars, and pigments—from food samples. This will enhance the sensitivity and accuracy of the assays. For instance, designing nanomaterials with specific adsorption capabilities can help selectively capture and remove interfering components while retaining target analytes. 2) Enhancement of detection limits and quantification accuracy: nanomaterials can serve as signal amplifiers to strengthen the detection signal. Additionally, incorporating multiple detection modes—for example, by combining electrochemical and optical methods—can help validate and complement the results from nanopore assays (Yu, Y. et al., 2024). 3) Construction of a high-throughput multi-parameter assay platform: developing a high-throughput parallel assay platform that integrates multiple nanopore arrays will enable the simultaneous processing of multiple samples or detection of various analytes.

## Summary and outlook

In summary, bio-nanopore technology has showcased a diverse range of applications in biomolecule detection, particularly in nucleic acid sequencing, protein identification, and small molecule analysis. Its exceptional sensitivity and accuracy have propelled advancements in nucleic acid sequencing technologies, facilitating applications such as whole genome sequencing and RNA modification detection. In the realm of protein analysis, bio-nanopore technology not only enables the identification of amino acids and their modifications but also advances single-molecule protein sequencing and peptide detection. Moreover, this technology effectively detects small molecule components in complex mixtures, including pharmaceutical compounds and environmental pollutants, manifesting its potential for innovation and application in the quality control of herbal medicines and the characterization of biological samples.

Future research should prioritize several key innovations and breakthroughs: 1) Optimization of materials and design: utilizing advanced genetic engineering techniques, natural biological nanopores (e.g., MspA, α-hemolysin) can be precisely engineered. Fine-tuning the structural features of nanopores can be achieved through genetic mutations and the insertion or deletion of gene fragments. Specifically, altering the number of specific amino acid residues within the pore allows for adjustments in pore size, thereby optimizing it to align with the size of target molecules and improving the assay's selectivity and sensitivity. Additionally, incorporating functional domains with specific recognition capabilities enables the nanopore to efficiently identify and capture particular types of biomolecules. Conversely, the elimination of unnecessary structural domains can reduce non-specific binding, enhancing the clarity and reliability of the detection signal. Additionally, the integration of novel nanomaterials (e.g., two-dimensional materials, quantum dots) with biological nanopores should be explored to develop composite nanopore structures that exhibit superior performance, further improving their detection capability and stability. 2) Enhancing detection efficiency and throughput: the construction of nanopore arrays using micro- and nanofabrication technologies, in conjunction with microfluidic chip platforms, allows for the optimization of sample flow paths and flow rates. This ensures that target biomolecules effectively and efficiently traverse the nanopore arrays in an orderly manner. Furthermore, the introduction of multi-channel parallel detection technology, alongside high-speed data acquisition and processing systems, enables simultaneous detection across multiple nanopores. 3) Reducing background noise and enhancing stability: in the chemical modification of the inner walls of nanopores, specific recognition molecules, such as antibodies and aptamers, are employed for targeted biomolecules (e.g., DNA, RNA, or proteins). These recognition molecules are securely bonded to the nanopore walls via covalent linkages (e.g., amide or thioester bonds) or electrostatic adsorption, ensuring high selectivity and affinity for the target biomolecules. Additionally, key parameters, including temperature, pH, and ion concentration in the detection environment, must be rigorously controlled to ensure the stability of the detection process and minimize the impact of environmental fluctuations on the results.

Looking ahead, the ongoing advancements in nanotechnology and bioengineering are poised to expand the applications of bio-nanopore technology across various domains including early disease diagnosis, personalized medicine, and environmental monitoring. This evolution is expected to significantly enhance the efficiency and accuracy of biomolecular detection and may also catalyze the development of innovative therapeutic strategies to address the increasing demand for medical and bioanalytical solutions.

## Data Availability

No datasets were generated or analyzed during the current study.
